# Assessing anxiety-linked impairment in attentional control without eye-tracking: The masked-target antisaccade task

**DOI:** 10.3758/s13428-022-01800-z

**Published:** 2022-03-15

**Authors:** Julian Basanovic, Jemma Todd, Bram van Bockstaele, Lies Notebaert, Frances Meeten, Patrick J. F. Clarke

**Affiliations:** 1 Centre for the Advancement of Research on Emotion, School of Psychological Science, The University of Western Australia, Perth, Australia; 2 School of Psychology, University of Sydney, Sydney, Australia; 3 Research Institute of Child Development and Education, University of Amsterdam, Amsterdam, Netherlands; 4 School of Psychology, University of Sussex, Brighton, UK; 5 Affective, Behavioural, and Cognitive Neuroscience Research Group, Curtin University, Perth, Australia

**Keywords:** Anxiety, Attentional control, Antisaccade

## Abstract

Contemporary cognitive theories of anxiety and attention processing propose that heightened levels of anxiety vulnerability are associated with a decreasing ability to inhibit the allocation of attention towards task-irrelevant information. Existing performance-based research has most often used eye-movement assessment variants of the antisaccade paradigm to demonstrate such effects. Critically, however, eye-movement assessment methods are limited by expense, the need for expert training in administration, and limited mobility and scalability. These barriers have likely led to researchers’ use of suboptimal methods of assessing the relationship between attentional control and anxiety vulnerability. The present study examined the capacity for a non-eye-movement-based variant of the antisaccade task, the masked-target antisaccade task (Guitton et al., 1985), to detect anxiety-linked differences in attentional control. Participants (*N* = 342) completed an assessment of anxiety vulnerability and performed the masked-target antisaccade task in an online assessment session. Greater levels of anxiety vulnerability predicted poorer performance on the task, consistent with findings observed from eye-movement methods and with cognitive theories of anxiety and attention processing. Results also revealed the task to have high internal reliability. Our findings indicate that the masked-target antisaccade task provides a psychometrically reliable, low-cost, mobile, and scalable assessment of anxiety-linked differences in attentional control.

## Introduction

Contemporary cognitive theories of anxiety and attention processing propose that heightened levels of anxiety vulnerability are associated with a decreased ability to inhibit the allocation of attention towards task-irrelevant information (Derakshan & Eysenck, 2009; Eysenck et al., 2007). This proposal has been supported by recent meta-analytic findings demonstrating that participants with elevated anxiety vulnerability demonstrate reduced attentional control performance as compared to individuals less vulnerable to anxiety (Shi et al., 2019). This phenomenon has been labelled an anxiety-linked impairment in attentional control.

A considerable body of literature examining this anxiety-linked impairment in attentional control has employed questionnaire measures to assess attentional control. In particular, the Attentional Control Scale developed by Derryberry and Reed (2002) has sought to measure attentional skills related to voluntary executive functions, using participants’ responses on this scale as an index of attentional control ability. In their original study, the researchers observed poorer self-reported attentional control ability among participants with greater levels of anxiety vulnerability. One benefit of such self-report measures is that they demonstrate a high level of internal reliability (Fajkowska & Derryberry, 2010; Ólafsson et al., 2011; Quigley et al., 2017) and are easily accessible and scalable in delivery (e.g. online delivery). Thus, the Attentional Control Scale is understandably favoured by researchers, and many investigators have employed this scale to investigate attentional control amongst participants who vary in anxiety vulnerability (Bardeen & Orcutt, 2011; Muris et al., 2004; Ólafsson et al., 2011; Schoorl et al., 2014). Critically, however, the use of questionnaire measures to index cognitive processing is limited due to their measurement of one’s *beliefs* about attentional control processes. Indeed, several recent studies have demonstrated no evidence of an association between participants’ self-reported level of attentional control ability and their level of performance on attentional control assessment tasks (Quigley et al., 2017; Reinholdt-Dunne et al., 2009, 2013; Todd et al., 2022; Williams et al., 2017). Thus, evidence that scores on the scale reflect one´s ability to control attention is limited.

In contrast to questionnaire measures, researchers have also employed performance-based measures to assess attentional control. A performance-based assessment that has been frequently employed by researchers investigating anxiety-linked differences in attentional control is the antisaccade paradigm. The antisaccade paradigm (Hallett, 1978) involves a series of trials that each present an abrupt distractor stimulus in a left or right peripheral location onscreen. Participants are required to inhibit the reflexive orienting of attention towards the distractor stimulus when it appears and to direct attention to the opposite screen position as rapidly as possible. More rapid movement of attention to the desired location and fewer erroneous attention movements towards the distractor stimulus are believed to represent better attentional control. When examining task performance amongst individuals who vary in anxiety vulnerability, investigators have observed that individuals with relatively heightened levels of anxiety vulnerability demonstrate poorer performance as compared to individuals with lower levels of anxiety vulnerability (Ansari & Derakshan, 2010, 2011; Basanovic et al., 2018; Derakshan et al., 2009; Myles et al., 2020; Wright et al., 2014). Further research has also demonstrated modulation of anxiety-linked differences in performance on the antisaccade task via the adjustment of the content of the distractor stimulus (Chen et al., 2014; Reinholdt-Dunne et al., 2012; Wieser et al., 2009). Thus, the antisaccade paradigm has demonstrated sensitivity to anxiety-linked differences in attentional control and has served as a useful tool in the investigation of anxiety-linked differences in the processing of neutral and emotional information.

At present, research focusing on the relationship between anxiety vulnerability and antisaccade performance has consistently assessed performance through the recording of eye movements. This approach provides researchers with the benefit of measuring the accuracy and latency of participants’ attentional movements across the duration of a trial. In addition, researchers have reported that eye-movement-based measures hold a high level of internal reliability (α_Cronbach_ = .85; Ettinger et al., 2003; *r*_(Spearman-Brown)_ = .95; Myles et al., 2020). However, such approaches come with practical limitations including financial barriers, the need for specialist operator training, and the inability to deploy assessments on a large scale.

Alongside assessments using eye-movement recording, researchers have developed approaches that instead utilise manual response latencies to index attentional control. For example, researchers have presented a visual target in the location opposite the distractor stimulus and recorded the speed at which participants are able to discriminate the identity of the target via a manual key-press (Ansari et al., 2008; Basanovic et al., 2017, 2021; Derakshan et al., 2009; Wright et al., 2014). Such tasks are readily accessible to researchers, require only minimal technical expertise to adopt, and can be easily completed on devices in-lab and through the internet. However, while these approaches resolve the practical issues inherent to eye-movement assessments, estimates of the internal reliability of these methods have demonstrated modest reliability (*r*_(Spearman-Brown)_ = .59; Basanovic et al., 2021), and researchers have not detected anxiety-linked impairment in attentional control when using these tasks.

Thus, the capacity for researchers to investigate the relationship between anxiety vulnerably and attentional control is presently impeded by the absence of accessible, scalable, and reliable performance-based assessment methods that have been demonstrated to be sensitive to anxiety-linked differences in attention control. One task that may help solve this problem was developed by Guitton et al. (1985), labelled herein the *masked-target antisaccade assessment task*. This task first presents a distractor stimulus followed by a visual target in an opposing screen location, and participants are required to discriminate the identity of the target. Importantly, the target is presented only briefly (150 ms) before being masked. Thus, accurate discrimination of the target necessitates rapid attentional movement away from the initial distractor stimulus. The task indexes performance via target discrimination accuracy, with a greater number of accurate responses across trials reflecting greater attentional control. Notably, this task has been shown to be associated with other tasks that require inhibition of pre-potent responses and cognitive control. For example, Friedman and Miyake (2004) and Miyake et al. (2000) observed that performance on the task was correlated with performance on Stroop and stop-signal tasks (*r* = .20 to .23 and *r* = .16 to .19 respectively), though it is notable that the sizes of these correlations also indicate the influence of unshared processes on measures from each task. In addition, Kane et al. (2001) and Unsworth et al. (2012) demonstrated that individuals with relatively greater working memory capacity across operation, reading, and symmetry span tasks exhibited better performance on the task as compared to individuals with lower working memory capacity (Unsworth et al, Cohen’s *d* = .62; Kane et al, Cohen’s *d* = .40). However, Roberts et al. (1994) found no association between performance on the task and performance on reading-span and counting-span variants of working memory capacity assessments. In addition to these associations, the task has been shown to hold a relatively high level of internal reliability (*r*_(Spearman-Brown)_ = .77 to .87; Friedman & Miyake, 2004; Miyake et al., 2000). Thus, the masked-target antisaccade assessment approach stands as a promising performance-based method for investigating anxiety-linked differences in attentional control that circumvents the limitations of eye-movement- and response-latency-based approaches.

Critically, no research to date has sought to directly investigate whether the masked-target antisaccade assessment task demonstrates sensitivity to anxiety-linked differences in attentional control. Thus, the aim of the present study was to determine whether the masked-target antisaccade assessment approach is sensitive to anxiety-linked differences in attentional control. The study recruited individuals to complete a questionnaire measure of anxiety vulnerability followed by a masked-target antisaccade assessment task. Analyses examined the association between anxiety vulnerability and performance on the task and the internal reliability of the task. It was predicted that if the task is sensitive to anxiety-linked differences in attentional control, then greater levels of anxiety vulnerability will be associated with poorer performance on the task.

## Method

### Participants

Recruitment of participants to this study was conducted online as part of the Cognition and Emotion Research Collaboration Initiative (CERCI) with researchers in Australia and England. In total, 342 individuals (243 female, 96 male, 3 non-binary; age *M* = 20.75 years, *SD* = 4.79 years) participated in the study. Invitations to participate were made available to students through undergraduate participant pools at Curtin University (*N* = 104), the University of Western Australia (*N* = 77), and the University of Sydney (*N* = 116), and via social media through the University of Sussex (*N* = 45).

### Materials

#### Depression, Anxiety, Stress Scale–Anxiety Scale

The measure of anxiety was drawn from the 21-item version of the Depression, Anxiety, Stress Scale questionnaire (DASS-21; Lovibind & Lovibond, 1995). The DASS-21 Anxiety Scale comprises seven self-report items that assess the degree to which participants experienced symptoms of anxiety during the previous week. Scores on the anxiety scale range from 0 to 21, with higher scores representing higher levels of anxiety. The DASS-21 has demonstrated high test-retest reliability and high concurrent and construct validity among university student and general community populations (Antony et al., 1998). Internal consistency for the DASS-21 Anxiety Scale in the current study sample was high (Cronbach´s alpha = .87).

#### Masked-target antisaccade assessment task

**Fig. 1:**
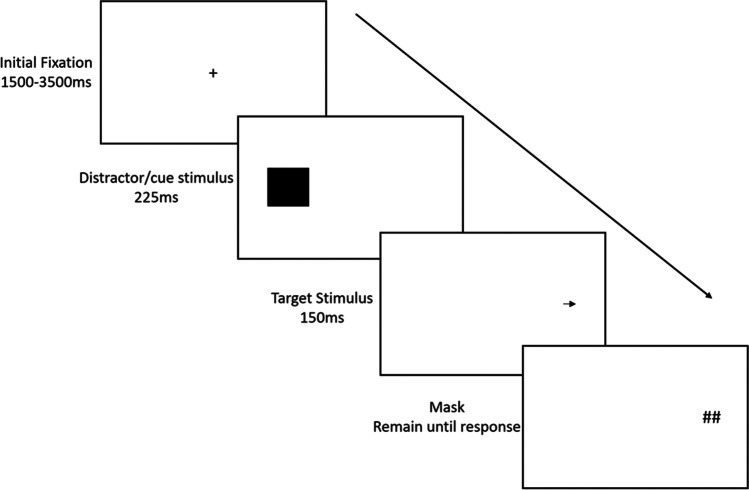
Schematic of the progression of a single trial presented in the masked-target antisaccade assessment task. Note: figure not to scale

The masked-target antisaccade assessment task was delivered to measure attentional control. The design of the task reflected that originally described by Roberts et al. (1994)^1^, and later adopted by Miyake et al. (2000) and Friedman and Miyake (2004). The task measures the ability of participants to execute rapid attentional movements under conditions that require the inhibition of attention to a peripherally presented distractor stimulus, to accurately discriminate the identity of a target presented in the opposite location. The task comprised 90 trials. A schematic representation of a trial is depicted in Fig. 1.

Each trial commenced with a small fixation cross presented in the centre of the screen for a duration between 1500 and 3500 ms, in increments of 250 ms, selected at random without replacement. Next, the fixation cross was removed and simultaneously the distractor cue stimulus was presented for 225 ms. The stimulus was a black square, 20 mm × 20 mm in size, and presented 85 mm horizontally to the left or right of the initial fixation cross with equal frequency across trials. Following this, a visual target was presented in the screen position opposite the cue for 150 ms. The target was a small black arrow, 5 mm in length, pointing left, right, or upward with equal frequency. The target arrow was masked with the icon ‘##’. Participants were required to report the direction of the target arrow by pressing the corresponding left, right, or up arrow key on their keyboard. Following the response, the screen was cleared, and the next trial began following a 500 ms inter-trial interval.

The brief exposure duration of the target is designed to ensure that successful discrimination of the cue identity requires participants to inhibit the execution of reflexive attentional movements towards the cue stimulus, to rapidly direct attention to the position of the target. Performance on the task is indexed via the number of correctly discriminated targets, such that a greater number of correctly discriminated targets indicates a greater level of attentional control.

The assessment task was preceded by a block of 20 practice trials. To familiarise participants with the speed of the task, practice trials presented the target arrow on screen for a gradually decreasing duration across trials (250 ms, to 200 ms, to 150 ms), and informed participants of any incorrect responses during the inter-trial interval.

### Procedure

Upon accepting an online invitation to participate in the study, participants accessed the study via an online link using their personal computer. All assessments were delivered via the Inquisit Web platform. Participants were first presented information on the requirements of the study and provided informed consent. Participants next provided demographic information before completing a set of questionnaire measures that included the DASS-21. Additional questionnaires were completed for separate research projects. Participants then completed the masked-target antisaccade assessment task, followed by additional cognitive assessments completed for separate research projects. The antisaccade assessment task was always delivered prior to delivery of other tasks, and was immediately preceded by detailed instructions on its requirements. The additional questionnaires and tasks did not include any other measure of attentional control, or any measure conceptually related to cognitive control. Upon conclusion of the cognitive tasks, participants received debriefing information. The duration of the entire study protocol was approximately 30 minutes.

## Results

### Participant exclusion and descriptive statistics

The need for a criterion that can differentiate between low levels of ability as compared to low task engagement or compliance is present in all tasks that use response accuracy as a dependent variable of interest. As such, a participant exclusion criterion was employed with the intention of excluding individuals whose low performance resulted from low task engagement or compliance, while retaining participants whose low performance reflected genuinely low ability. This criterion required participants to demonstrate correct responses on more than 40% of trials (36 correct responses) to be included in data analysis. Accuracy above this threshold holds less than 5% probability of occurring in the case of random responding on the task as per a binomial distribution, and so was considered an appropriate criterion for exclusion^2^.

Seventy-seven participants demonstrated a proportion below this threshold (range, 21–40%). Thus, subsequent statistical analysis was conducted on the remaining 265 participants. Descriptive statistics of demographic measures, DASS-Anxiety Scale scores, and the proportion of trials with correct responses on the antisaccade assessment task are present in Table 1.

**Table 1:** Descriptive statistics of demographic measures and measures of the proportion of correct responses on the antisaccade assessment task

Measure	Female	Male	Non-binary
Gender	184	78	3
	Mean (*SD*)		Range
Age (years)	20.79 (4.99)		17–58
DASS-21 – Anxiety Subscale	5.40 (4.76)		0–20
Antisaccade assessment task (proportion of responses correct)	.66 (.13)		.41–.97

*N* = 265

### Internal reliability of the masked-target antisaccade assessment task

The internal reliability of participants’ responses on the masked-target antisaccade assessment task was computed using a multi-permutation split-half correlation approach, which computed correlations between the proportion of correct responses across 5000 randomly generated split-halves (Parsons, 2021). The resulting Spearman-Brown corrected internal reliability estimate of the task was *r*_(Spearman-Brown)_ = .86, CI_95%_[.84–.88]. This indicated a high level of internal reliability for the measure.

### Association between anxiety and performance on the masked-target antisaccade assessment task

To determine the simple association between participants’ level of anxiety vulnerability and performance on the masked-target antisaccade assessment task, the correlation between *DASS-Anxiety Scale scores* and the *proportion of trials with correct responses* on the task was computed. This revealed a significant association between the two measures, *r*_(263)_ = −.15, *p* = .016, indicating that elevated levels of anxiety vulnerability were associated with a lower proportion of correct responses on the masked-target antisaccade assessment task.

To determine whether participants’ level of anxiety vulnerability predicted performance on the masked-target antisaccade assessment task, a logistic regression model was computed. The regression model included *proportion of trials with correct responses* as the dependant variable and *DASS-21 Anxiety Scale scores* as the predictor variable. The results of the regression analysis are present in Table 2.

**Table 2:** Results of the logistic regression model predicting number of correct responses in the masked-target antisaccade assessment task

Predictors	Response correct		
	Odds ratio	CI (95%)	*p*
(Intercept)	2.144	2.059 – 2.234	<.001*
DASS-21 Anxiety Scale Score	0.982	0.977 – 0.988	<.001*
N = 265			

The results revealed a significant effect of DASS-21 Anxiety Scale scores, such that greater DASS-21 Anxiety Scale scores predicted reduced performance on the antisaccade assessment task. Specifically, the results demonstrated that with every one-point increase in the score on the DASS-21 Anxiety Scale, the odds of a correct response were reduced by 1.8%. In addition, the model’s prediction of the proportion of correct responses for each possible score on the DASS-21 was computed. This revealed a decline in the proportion of trials with correct responses between individuals scoring 0 (predicted proportion = .68) and individuals scoring 21 (predicted proportion = .60) on the DASS-21 Anxiety Scale.

Readers interested in the association between performance on the antisaccade assessment task and measures of depression or stress recorded from the DASS-21 are encouraged to explore the data available at the public repository.

## Discussion

The aim of the present study was to determine whether the masked-target antisaccade assessment task is sensitive to anxiety-linked differences in attentional control and to assess the task’s internal reliability. The results revealed that greater levels of anxiety vulnerability predicted declining levels of performance on the task. This association is consistent with predictions made by contemporary theories of anxiety-linked differences in attentional processing (Eysenck & Derakshan, 2011) and is consistent with patterns of anxiety-linked impairment in attentional control observed by studies using eye-tracking variants of the antisaccade task (Ansari & Derakshan, 2010, 2011; Basanovic et al., 2018; Derakshan et al., 2009; Myles et al., 2020). Thus, the findings of the present study indicate that the masked-target antisaccade assessment task represents a methodology that is sensitive to anxiety-linked impairment in attentional control.

The analyses revealed that the magnitude of the simple association between performance on the masked-target antisaccade task and levels of anxiety vulnerability was small (*r*_(263)_ = −.15). Previous research examining the association between anxiety vulnerability and antisaccade performance using eye-tracking measures has demonstrated larger effect sizes (Basanovic et al., 2018; Wright et al., 2014; for a review of published effect sizes, see Shi et al., 2019). While larger effects may be expected given the predominant use of between-group designs across these studies, it is important for researchers to consider that the association demonstrated in the present study may require a relatively larger number of participants to detect reliably as compared to antisaccade tasks that employ eye-tracking measures.

The present study also revealed that the masked-target antisaccade task holds a high level of internal consistency in its measurement (*r*_(Spearman-Brown)_ = .86). Though the observed level of internal reliability is lower than some levels reported for eye-movement measures recorded during the antisaccade task (*r*_(Spearman-Brown)_ = .95; Myles et al., 2020), the present task demonstrated much greater reliability as compared to existing approaches used to measure antisaccade performance without eye-movement recording (*r*_(Spearman-Brown)_ = .59; Basanovic et al., 2021). Researchers have noted the constraints of poor or unknown psychometric reliability of cognitive assessment measures (Parsons et al., 2019), and have increasingly called for experiment designs that overcome these limitations. The present findings indicate that the masked-target antisaccade task represents a psychometrically reliable measurement option that can enable investigators to meet these challenges when investigating anxiety-linked differences in attentional control.

In addition to literature concerning the relationship between attentional control and anxiety vulnerability, a significant body of work concerns the relationship between attentional control and depression vulnerability (Snyder, 2013). This research includes the use of antisaccade assessment measures to index depression-linked differences in attentional control (Ainsworth & Garner, 2013). A post hoc analysis of the association between DASS-21 Depression Scale scores and antisaccade performance in the current study revealed a significant association, *r*_(263)_ = −.14, *p =* .025. Thus, the task may also hold benefits for researchers seeking to investigate the relationship between depression and antisaccade performance without employing eye-tracking. Future research could usefully examine the sensitivity and reliability of the task in demonstrating this association by replicating the design of the present study but with the aim of examining whether performance on the masked-target antisaccade task is associated with individual differences in depression vulnerability.

Though previous studies have demonstrated that performance on the masked-target antisaccade task is associated with other measures of cognitive control, supporting its validity as a measure of attention control (Friedman & Miyake, 2004; Miyake et al., 2000), researchers have not compared participants’ performance on the masked-target antisaccade task with their performance on eye-movement assessment variants of the antisaccade task. Thus, the degree to which measures on each task are convergent across individuals who vary in anxiety vulnerability is unclear. Future research could determine the convergence of these measures by assessing the association between performance on each task amongst individuals who vary in anxiety vulnerability. To the extent that the measures are associated with one another and predict common variance in anxiety vulnerability, this would support the possibility that the tasks are sensitive to the same anxiety-linked difference in attention control.

The present study delivered the same design as has been demonstrated to be associated with performance on other tasks believed to load on the construct of attentional/cognitive inhibition control (e.g. Miyake et al., 2000). Nonetheless, it is noteworthy that some researchers have also included assessment of performance under conditions that require the execution of ‘prosaccade’ attentional movements, where minimal control of attentional inhibition is required (Roberts et al., 1994). Accounting for prosaccade performance when indexing antisaccade performance aims to control for extraneous variables that may confound a relationship of interest, such as variation in visual processing speed, target discrimination speed, or ocular-motor movement speed. It is noteworthy, however, that when using the masked-tasked antisaccade task amongst non-specific participants, researchers have typically observed performance on these trials to be close to maximum levels with relatively little variation between participants (e.g. *M* = .96, *SD* = .04; Roberts et al., 1994). With respect to anxiety-linked differences in antisaccade performance, current evidence indicates that trait anxiety is associated specifically with variability in inhibition control, rather than visual processing speed, target discrimination speed, or ocular-motor movement speed (Ansari & Derakshan, 2010). Nonetheless, researchers may consider assessing prosaccade performance if adapting the masked-target antisaccade task for their own research, particularly where participant samples or assessment conditions are likely to result in variation in extraneous processes that may impact task performance.

Lastly, researchers may wish to note implications arising from the relatively large number of participants whose low task performance resulted in their exclusion from analysis in the present study. One implication derives from the fact that the present study recruited a relatively young and educated sample, which suggests that greater numbers of participants may be excluded if the same exclusion criteria are adopted amongst participant samples for whom attentional control ability is lower. Additionally, researchers will need to account for the exclusion rate when determining the number of participants to be recruited in their own studies, particularly when studies are conducted in contexts that permit low engagement or compliance, such as online. Changes to study design may permit more specific criteria that reduce exclusion rates. For example, prolonging the presentation duration of the target stimulus may result in a greater number of participants who are able to achieve a level of performance sufficient to avoid exclusion. Alternatively, including measures that directly assess task engagement and compliance may allow specific identification of participants to be excluded from analyses. Researchers adopting the masked-target antisaccade task may wish to consider whether such approaches may benefit their study.

For the moment, however, the present findings indicate that the masked-target antisaccade task provides a psychometrically reliable, low-cost, mobile, and scalable assessment of anxiety-linked differences in attentional control. It is hoped that the findings will aid researchers by affording a means to overcome existing barriers to robustly investigating anxiety-linked individual differences in attentional control, and so will facilitate research into this relationship into the future.

## Data Availability

Data and materials relating to the study reported in the manuscript are available at: https://www.osf.io/mc7zg/

## Footnotes

1 Unlike the present study, Miyake et al. (2000), or Friedman and Miyake (2004), Roberts et al. (1994) included a prosaccade trial condition. However, performance on this condition was very high across participants and showed small variance between participants. As such, this condition was not included in the present design.

2 It is worth noting that performance below this criterion for any participant does necessarily indicate low task engagement or compliance and may still reflect genuinely low ability. While we believe the current criterion strikes an appropriate balance between data inclusion and exclusion, other researchers may prefer alternative criteria.
